# Two-dimensional mesoporous metals: a new era for designing functional electrocatalysts

**DOI:** 10.1039/d3sc04244h

**Published:** 2023-10-25

**Authors:** Hao Lv, Ben Liu

**Affiliations:** a Key Laboratory of Green Chemistry and Technology of Ministry of Education, College of Chemistry, Sichuan University Chengdu 610064 China ben.liu@scu.edu.cn; b School of Chemistry and Chemical Engineering, Shanghai Jiao Tong University Shanghai 200240 China

## Abstract

Two-dimensional (2D) mesoporous metals contribute a unique class of electrocatalyst materials for electrochemical applications. The penetrated mesopores of 2D mesoporous metals expose abundant accessible undercoordinated metal sites, while their 2D nanostructures accelerate the transport of electrons and reactants. Therefore, 2D mesoporous metals have exhibited add-in structural functions with great potential in electrocatalysis that not only enhance electrocatalytic activity and stability but also optimize electrocatalytic selectivity. In this Perspective, we summarize recent progress in the design, synthesis, and electrocatalytic performance of 2D mesoporous metals. Four main strategies for synthesizing 2D mesoporous metals, named the CO (and CO container) induced route, halide ion-oriented route, interfacial growth route, and metal oxide atomic reconstruction route, are presented in detail. Moreover, electrocatalytic applications in several important reactions are summarized to highlight the add-in structural functions of 2D mesoporous metals in enhancing electrochemical activity, stability, and selectivity. Finally, current challenges and future directions are discussed in this area. This Perspective offers some important insights into both fundamental investigations and practical applications of novel high-performance functional electrocatalysts.

## Abstract 2

This Perspective summarizes recent progress in the synthesis and electrocatalytic applications of two-dimensional mesoporous metals and thus ensures a new era for designing high-performance functional electrocatalysts.

## Graphical abstract

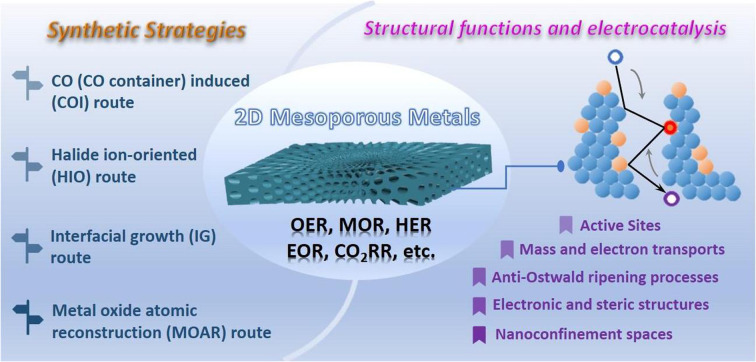

## 1. Introduction

Metal nanostructures feature unique physicochemical properties and have evoked their wide utilization in catalysis and electrocatalysis.^1–9^ The proper d-band electronic structure of metal nanostructures ensures moderate chemisorption strengths of the reactants/electrons, while their nanostructures expose more metal active sites.^1,10–16^ Of various metal nanostructures available, mesoporous metals composed of abundant and highly penetrated 2–50 nm pores have been paid gradually increasing attention due to their various applications.^17–25^ On the one hand, concave/convex mesochannels enlarge the utilization efficiency of metals and produce abundant undercoordinated high-index metal sites, which thus enhances their (electro)catalytic activity.^26–36^ Meanwhile, continuous metal frameworks accelerate the transport of electrons and reactants and inhibit the physical Ostwald ripening process, which increases the (electro)catalytic activity and synergistically enhances the stability.^37–41^ On the other hand, mesoporous metals provide a remarkable nanoconfinement environment that enriches the reaction intermediates within mesopores and optimizes their (electro)catalytic selectivity.^42–49^ However, the longer mesochannels of three-dimensional (3D) mesoporous metal nanoparticles remarkably decelerate the transport of reactants/products and competitively result in a huge wastage of metal sites in (electro)catalysis.

**Fig. 1:**
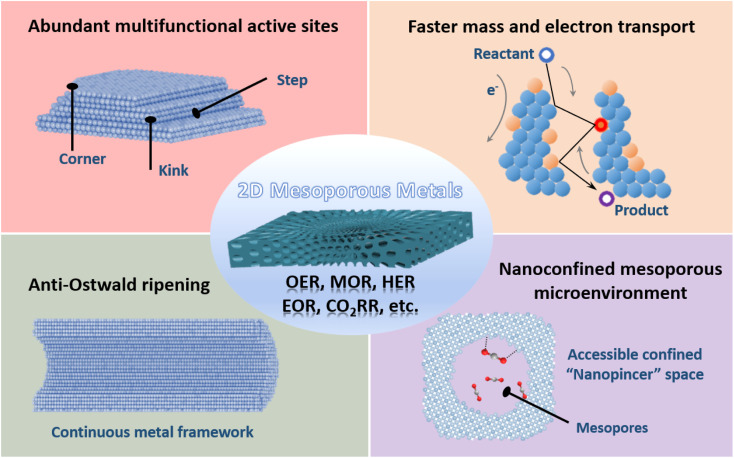
Structural functions of 2D mesoporous metals for utilization in electrocatalysis.

Further, nanostructuring mesoporous metals into 2D anisotropic nanostructures is an efficient and highly promising route to enlarge the utilization efficiency of exposed metal active sites and maximize their electrocatalytic performance.^50–53^ 2D mesoporous metals that subtly combine penetrated mesoporosity and 2D nanostructures into one unique material hold add-in structural functions, including higher (electro)catalytically active sites, faster transport of electrons and reactants within the active mesochannels, and a well-defined nanoconfined mesoporous microenvironment (Fig. 1). These structural advantages have highlighted the applications of 2D mesoporous metals that are more active, stable, and selective in various electrocatalytic reactions. More importantly, unique 2D mesoporous metal nanostructures are expected to amplify their physicochemical properties distinct to their counterpart nanostructures and to explore new utilizations from catalysis to bio-related applications. However, the synthesis of 2D mesoporous metals is remarkably complicated and requires a precise control over their thermodynamic and kinetic processes for an anisotropic epitaxial growth of 2D mesoporous metals along the mesopore-forming templates. Besides, other reaction conditions should be carefully regulated to tune their physical parameters, including crystalline phase, crystallinity, mesoporous structure, framework thickness, *etc.*

In this Perspective, we present the latest developments in synthesizing 2D mesoporous metals and exploring their important utilizations in electrocatalysis. We first summarize the current synthetic strategies of 2D mesoporous metals with controlled crystallinity and mesoporosity. Their formation procedures and mechanisms are also discussed in detail. We then present the add-in structural functions of 2D mesoporous metals in enhancing their electrocatalytic performance (activity, stability, and selectivity). We finally provide a brief summary of 2D mesoporous metals and more importantly offer an outlook on the future developments in this area. We expect the insights summarized in this Perspective may help the readers in finding their respective solutions of 2D mesoporous metals. However, this Perspective does not focus on the synthesis and applications of ultrathin 2D metal dendrites. The readers are encouraged to refer to recent reviews and research papers for more details.^54–56^

## 2. Synthetic strategies of 2D mesoporous metals

In this section, we systemically introduce four main synthetic strategies, including the CO (and CO container) induced (COI) route, halide ion-oriented (HIO) route, interfacial growth (IG) route, and metal oxide atomic reconstruction (MOAR) route, for preparing 2D mesoporous metals with well-defined nanostructures as follows.

### 2.1 CO (and CO container) induced (COI) strategy

CO and their corresponding containers are important for their strong binding interactions on the basal (111) facets of metals and thus enable the epitaxial growth of 2D anisotropic metal nanocrystals.^57^ Despite some encouraging progress over recent years, 2D metals with abundant penetrated mesopores (2D mesoporous metals) are still rarely reported, mostly because of the difficulty in controlling thermodynamic and kinetic processes during the nucleation of 2D mesoporous metals along the mesopore-forming templates.

**Fig. 2:**
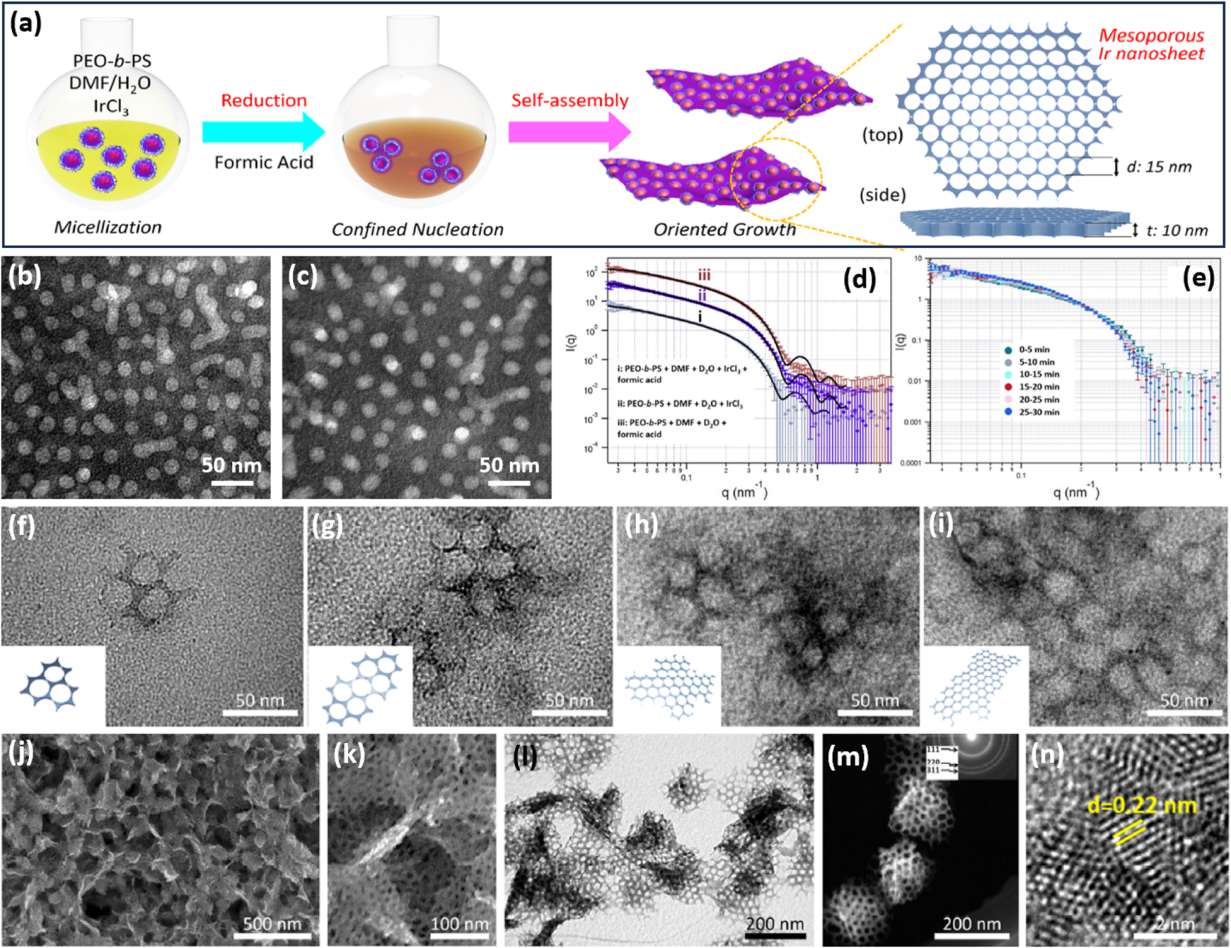
COI strategy. (a) A scheme illustrating the synthetic process of 2D mesoporous Ir nanosheets by a COI method. TEM images of polymeric micelles (b) before and (c) after the addition of formic acid and IrCl_3_. SANS patterns of a polymeric micelle at room temperature with (d) different agents and (e) different reaction times. (f–i) TEM images of mesoporous Ir nanosheets with different reaction times. (j) Low-magnification SEM image, (k) high-magnification SEM image, (l) TEM image, (m) HAADF-STEM image, and (n) high-resolution TEM image of mesoporous Ir nanosheets. Reproduced from ref. 50 with permission from the American Chemical Society, Copyright 2018.

In a pioneer work, Yamauchi *et al.* reported the first example of preparing 2D mesoporous iridium (Ir) nanosheets with a penetrated mesoporosity by a facile COI synthetic strategy (Fig. 2a).^50^ The authors first prepared uniform spherical polymeric micelles by the self-assembly of poly(ethylene oxide)-*block*-polystyrene (PEO-*b*-PS) in a dimethylformamide (DMF) and H_2_O mixed solution (Fig. 2b). After the addition of an Ir precursor, Ir^3+^ bonded with the PEO block of polymeric micelles by the Columnar and coordination interactions (Fig. 2c). These metal/polymer micelles were structurally stable, which did not change in the presence of a metal precursor and reducing agent. The stabilized micelles had been clearly confirmed by small-angle neutron scattering (SANS) (Fig. 2d). Meanwhile, spherical micelles were also stable during the nucleation and growth of Ir nanocrystals (Fig. 2e). These stabilized micelles behaved as the mesopore-forming template and were crucial to the formation of mesoporous Ir nanocrystals. After that, formic acid (HCOOH) as the reducing agent was injected into the above solution to direct the nucleation of Ir nanocrystals. Different to other reducing agents, HCOOH was degraded into carbon monoxide (HCOOH → CO + H_2_O), which was strongly bound to the basal (111) facets of Ir and caused an epitaxial growth of 2D nanosheets with anisotropic nanostructures.^58^ In this step, stabilized metal/polymer micelles were further assembled into 2D nanosheets and formed spherical mesopores. In sharp contrast, in the presence of other reducing agents, only spherical mesoporous structures with a lower surface energy were prepared accordingly. These results highlighted that the formation of 2D mesoporous Ir nanosheets was the result of a precise control over anisotropic epitaxial growth with PEO-*b*-PS as the mesopore-forming template and HCOOH (CO) as the reducing and structure-directing agent.

Transmission electron microscopy (TEM) images collected at different reaction times exhibited the in-plane epitaxial growth of 2D mesoporous Ir nanosheets. As shown in Fig. 2f–i, very tiny nanocrystals were formed in the initial stage. After further fusing together, mesoporous Ir nanocrystals became bigger along the plane orientations and anisotropically grew to form 2D mesoporous nanosheets. Low-magnification scanning electron microscopy (SEM) and TEM images showed that the final products were highly uniform and free-standing with a 2D nanosheet morphology and penetrated mesoporous structure (Fig. 2j–l). Mesopores were periodically ordered with a hexagonal packing mode (Fig. 2m). High-resolution TEM images demonstrated that the lattice fringe of the wall framework was 0.22 nm, which belongs to the (111) plane of a face cubic centered (fcc) Ir nanocrystal. Subsequently, the authors further explored 2D mesoporous Ir nanosheets as the template for preparing other Ir-based mesoporous metal nanomaterials, for example, mesoporous dual-phase NiB and Ir heterostructures.^59^ These 2D mesoporous metals exposed more catalytically active sites and accelerated electron/mass transport, thus remarkably enhancing their performance in (electro)catalysis (discussed later).

### 2.2 Halide ion-oriented (HIO) strategy

**Fig. 3:**
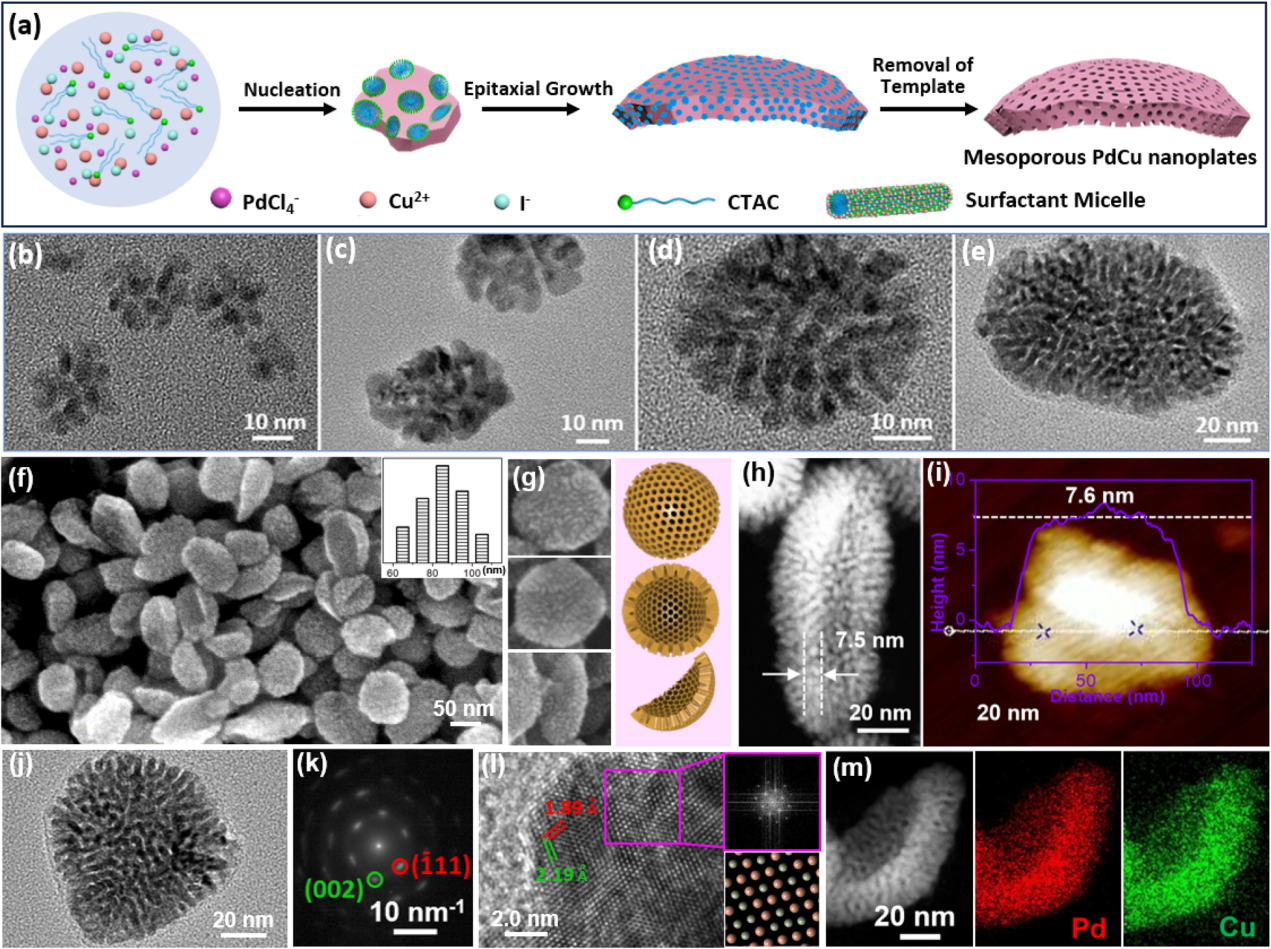
HIO strategy. (a) Schematic illustration of the synthetic process of 2D mesoporous Pd-based nanoplates by a HIO route. (b–e) Time-dependent TEM images of 2D PdCu single-crystalline mesoporous nanoplates during the epitaxial growth process. (f, g) SEM and (h) HAADF-STEM images, (i) atomic force microscope (AFM) image, (j) TEM image and (k) corresponding SAED pattern, and (l) high-resolution TEM image and corresponding FFT pattern (purple square area), and (m) HAADF-STEM EDX elemental mapping images of 2D PdCu single-crystalline mesoporous nanoplates. Reproduced from ref. 51 with permission from Wiley, Copyright 2022.

As well as CO and its containers, halide ions can selectively bind on typical crystalline facets of metal nanocrystals and direct the formation of 2D nanostructures.^60,61^ Our group thus extended the HIO strategy for the formation of 2D mesoporous metals in the presence of a mesopore-forming surfactant (Fig. 3a).^51^ The synthesis relied on amphiphilic cetyltrimethylammonium chloride (CTAC) as the surfactant and extra I^−^ as the structure-selective agent. In a typical synthesis of 2D mesoporous PdCu nanoplates, the metal precursors were mixed in an aqueous solution containing predominantly CTAC and I^−^. In this synthesis, the extra I^−^ strongly bind on the (100) facets of fcc Pd(Cu), while quaternary group of CTAC favorably adsorb on their (110) facets. After the injection of ascorbic acid (AA) that behaved as the reducing agent, anisotropic epitaxial growth with CTAC along the (110) facets produced 2D mesoporous metal nanoplates. Meanwhile, extra I^−^ also optimized the reduction potentials of PdCl_4_^2−^ (by PdI_4_^2−^) and ensured the precise nucleation and preparation of single-crystalline structure. Besides, the nucleation and epitaxial growth of fcc Pd(Cu) on uncovered (111) facets slightly curved the 2D mesoporous metals and formed high-curvature nanoplates.

Epitaxial growth of 2D single-crystalline mesoporous PdCu nanoplates was confirmed by time-dependent TEM observations collected under different reaction times. Clearly, tiny porous nanocrystals were formed immediately after the addition of AA (Fig. 3b) and gradually grew epitaxially along the (110) facets with a CTAC template into highly uniform 2D mesoporous PdCu nanoplates with a single-crystallinity and curved nanostructure (Fig. 3c–e). In comparison with spherical mesopores formed by PEO-*b*-PS, CTAC self-assembled into cylinder micelles and thus produced wormlike mesoporous channels. SEM and TEM images showed that the HIO strategy was powerful for a controllable preparation of high-quality curved single-crystalline mesoporous PdCu nanoplates (Fig. 3f–i). Meanwhile, there were abundant mesopores that radially penetrated the nanoplates. Moreover, the products were very thin with an average shell height of only 7.6 nm. The selected area electron diffraction (SAED) pattern of a single nanoplate showed a nearly single set of bright spots, corresponding to (110) facet exposed single-crystalline nanocrystals (Fig. 3j and k). A similar result was also confirmed by the high-resolution TEM images and corresponding fast Fourier transform (FFT) patterns (Fig. 3l). In addition to uniform metal distributions (Fig. 3m), the results highlighted the successful synthesis, for the first time, of 2D mesoporous metals with single-crystallinity and a curved nanostructure. Considering the diversity of halide ions on the selective metal crystalline facets,^7,10,62^ the HIO strategy would open a blue ocean for designing and synthesizing novel 2D mesoporous metals with well-desired functional metal compositions.

### 2.3 Interfacial growth (IG) strategy

**Fig. 4:**
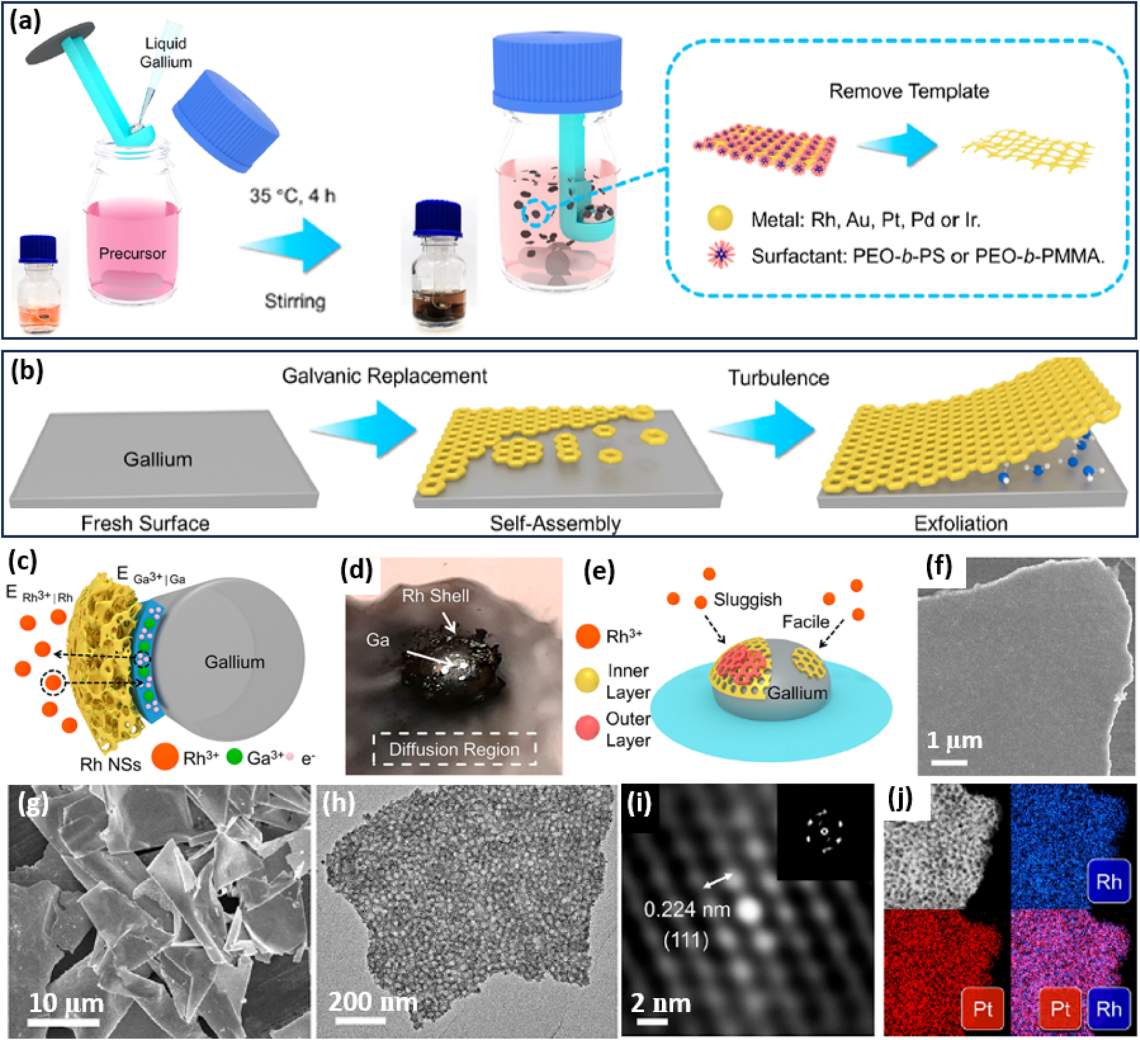
IG strategy. Schematic illustration of the synthetic process of 2D mesoporous metals by a liquid metal IG route at the (a) macroscopic and (b) microscopic levels. (c) Schematic illustration for ion permeation and electronic transfer during the liquid metal IG synthesis on a Ga droplet. (d) Photograph of a Ga droplet in the precursor solution on water glass. (e) Schematic representation of the relationship between the reduction rate and thickness of a mesoporous Rh layer. (f) SEM image of the top view of an individual mesoporous Rh nanosheet. (g) Low-magnification SEM and (h) TEM images, (i) Fourier-filtered lattice fringe and corresponding FFT pattern, and (j) HAADF-STEM elemental mapping images of 2D mesoporous RhPt nanosheets. Reproduced from ref. 64 with permission from the American Chemical Society, Copyright 2022.

2D metals and/or oxides can be also formed on a sacrificial 2D template, for example, liquid metals,^63^ by precisely adjusting the interfacial interactions between the metal precursors and templates. Followed by the IG route, an excellent work reported by Wang *et al.* found that 2D mesoporous metals can be selectively grown on the interface of a sacrificial liquid metal, gallium (Ga) (Fig. 4a and b).^64^ In the synthesis, PEO-*b*-PS or poly(ethylene oxide)-*block*-poly(methyl methacrylate) (PEO-*b*-PMMA) was utilized as the soft template to form spherical mesopores. Typically, the authors mixed the unique liquid metals into an aqueous solution containing metal precursors and PEO-*b*-PS/PEO-*b*-PMMA. The metal precursors were quickly moved to the interface of liquid metal (Ga). Then, due to the different reduction potentials of Ga and other metals, the galvanic replacement reactions inevitably happened between Ga and the metal precursors, resulting in a self-limiting anisotropic synthesis of 2D mesoporous metals on a robust Ga surface in the presence of the mesopore-forming surfactant (PEO-*b*-PS or PEO-*b*-PMMA) (Fig. 4c). The galvanic replacement reaction was directly observed from the photo of the Ga droplet in the reaction solution (Fig. 4d). Meanwhile, the galvanic replacement reaction favored the direct interface of the Ga droplet, resulting in the favorable formation of tightly covered shells instead of multi-shell frameworks (Fig. 4e). In this stage, the lower nucleation rate in the absence of other reducing agents kinetically facilitated the anisotropic epitaxial growth of a planar nanostructure with a flat surface. Finally, 2D mesoporous metal nanoplates were accordingly obtained by exfoliating from the Ga surface and removing the mesopore-forming surfactant (Fig. 4f).

Based on the liquid metal IG strategy, the authors successfully prepared some 2D mesoporous metal nanosheets with a high homogeneity and purity (Fig. 4g). The lateral length of their mesoporous PtRh nanosheets was as large as 10 μm with an average thickness of 29 nm. A high-magnification TEM image further showed abundant spherical mesopores through the nanosheets (Fig. 4h). The Fourier-filtered lattice fringe and corresponding FFT pattern as well as HAADF-STEM elemental mapping images confirmed that 2D mesoporous nanosheets were compositionally alloyed (Pt/Rh), indicating the precise controllability in preparing both monometallic and multimetallic alloys (Fig. 4i and j). As a result, 2D mesoporous metal nanosheets, including Rh, Au, Pt, RhPt, and RhPdIrPtAu, were successfully prepared by the liquid metal IG strategy. The authors thus expected that the liquid metal IG route can introduce a library of 2D mesoporous metals with controlled compositions on various sacrificial templates for their wide utilization in (electro)catalysis. Besides, 2D mesoporous Pt nanosheets with well-ordered mesostructures have also been prepared through the silicon IG route by spin-coating the precursor solutions containing PtCl_4_^2−^ and spherical micelles assembled by poly(styrene-*block*-2-vinyl pyridine-*block*-ethylene oxide) (PS-*b*-P2VP-*b*-PEO) on a silicon substrate.^65^

### 2.4 Metal oxide atomic reconstruction (MOAR) strategy

**Fig. 5:**
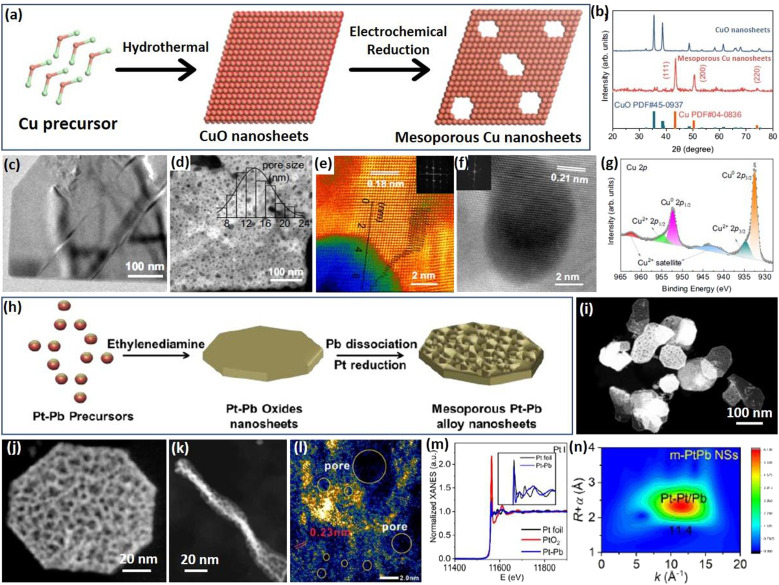
MOAR Strategy. (a) Schematic illustration of preparing 2D mesoporous Cu nanosheets by the MOAR route. (b) XRD patterns of CuO nanosheets and mesoporous Cu nanosheets. (c) TEM image of CuO nanosheets. (d) HAADF-STEM image, (e, f) atomic-resolution HAADF-STEM images, and (g) high-resolution XPS Cu 2p spectrum of mesoporous Cu nanosheets. Reproduced from ref. 52 with permission from Springer Nature, Copyright 2021. (h) Schematic illustration of preparing 2D mesoporous PtPb nanosheets. (i and j) Low-magnification HAADF-STEM images, (l) high-magnification TEM image, (k) TEM image (side view), (m) XANES spectrum and (n) corresponding MT plots of mesoporous PtPb nanosheets. Reproduced from ref. 53 with permission from Wiley, Copyright 2023.

Apart from the above template synthesis, the template-free route has also been reported to prepare 2D mesoporous metals by a precise atomic reconstruction of their metal oxide counterparts. The synthesis divided generally into a two-step process, including the preparation of 2D metal oxide nanosheets and the reduction (atomic reconstruction) into 2D mesoporous metal nanosheets. Followed by the MOAR route, a typical example reported recently by Huang *et al.* prepared 2D mesoporous Cu nanosheets by an *in situ* electrochemical reduction of 2D CuO nanosheets (Fig. 5a).^52^ The authors first prepared ultrathin 2D CuO nanosheets through a simple hydrothermal route in a Teflon-lined autoclave. Then, the CuO nanosheets were utilized as the template and directly converted into the reduced mesoporous Cu nanosheets under a typical CO_2_ reduction flow-cell electrolyzer using K_2_SO_4_ as the electrolyte and under a galvanostatic mode for 1 h. The atomic reconstruction during the release of O atoms produced abundant voids and thus formed randomly dispersed mesopores without destroying the nanosheet nanostructure. Wide-angle XRD patterns confirmed the crystalline phase transformation (or atomic reconstruction) from CuO to Cu (Fig. 5b). In comparison to the Cu counterparts that had a smooth nonporous surface (Fig. 5c), the reduced Cu nanosheets were composed of abundant mesopores that uniformly dispersed and penetrated in the nanosheets (Fig. 5d). The mesopore distribution of 2D mesoporous Cu nanosheets was slightly broad in the range of 8–24 nm. Besides, penetrated mesopores of the mesoporous Cu nanosheets were further confirmed by the brightness contrast and height profile obtained from TEM images (Fig. 5e). Moreover, high-resolution TEM images and corresponding FFT patterns, as well as X-ray photoelectron spectroscopy (XPS), showed the formation of 2D Cu nanocrystals in the metallic state rather than the oxide state (Fig. 5f and g), further highlighting the MOAR route to synthesize 2D mesoporous metals. Similarly, 2D mesoporous Bi nanosheets have been prepared by the atomic reconstruction of Bi(OH)_3_ nanosheets under electrocatalytic conditions.^66^

A similar work developed by Dai *et al.* was to prepare 2D bimetallic mesoporous PtPb alloy nanosheets by atomic reconstruction of Pt–Pb oxide nanosheets (Fig. 5h).^53^ The synthesis was carried out as a hydrothermal reaction in DMF solution containing Pt(acac)_2_, Pb(acac)_2_, ethylenediamine, and polyvinylpyrrolidone (PVP) at 145 °C in a pressure vessel. During the synthesis, 2D Pt–Pb oxide nanosheets were first formed in the presence of alkaline ethylenediamine. Then, metallic Pt–Pt bonds were produced gradually by the reduction of Pt oxides, but Pb remained in the oxidized state. As the reaction proceeded, oxidized PbO species were also dissociated into metallic Pb and further alloyed with Pt into bimetallic PtPb nanosheets. Meanwhile, the atomic reconstruction of metal oxide nanosheets into metals formed the abundant voids and finally synthesized the 2D mesoporous PtPb nanosheets. An HAADF-STEM image demonstrated that the products synthesized by the MOAR route were composed of ultrathin nanosheets with polygonal nanostructures (octagons and hexagons) (Fig. 5i and j). Meanwhile, the nanosheets were very thin with an average thickness of 8.0 nm (Fig. 5k). Abundant mesopores of 3.3 nm penetrated into the nanosheets, which thus exposed more active metal sites (Fig. 5l). The authors also performed an X-ray adsorption near edge structure (XANES) study, in which mesoporous PtPb alloy nanosheets disclosed a similar spectrum to that of Pt foil (but different to that of PtO_2_), indicating they were in the metallic state (Fig. 5m). Besides, wavelet transform (WT) plots of the mesoporous PtPb nanosheets showed a lower *k* value of 1.14 nm, confirming the Pt–Pb(PbO) interface, further confirming the template-free atomic reconstruction of metal oxide nanosheets to synthesize 2D mesoporous metals (Fig. 5n). These results thus enriched the MOAR route to prepare 2D multimetallic mesoporous nanosheets for various electrocatalytic reactions.

## 3. Electrocatalytic performance of 2D mesoporous metals

In this section, we summarize the electrocatalytic performance, including activity and stability as well as selectivity, of 2D mesoporous metals in electrocatalysis based on their multiple structural functions, as follows.

### 3.1 Enhanced electrocatalytic activity and stability of 2D mesoporous metals

2D mesoporous metals ensure multiple structural functions, which have been widely applied as highly active and stable electrocatalysts. On the one hand, both concave and convex mesopores ensured a high surface curvature of the crystalline metal sites. These abundant mesopores not only increased the numbers of exposed metal sites but also produced more undercoordinated and high-index active sites, which remarkably enhanced the electrocatalytic activity of 2D mesoporous metals. On the other hand, 2D nanostructures and continuous metal frameworks kinetically accelerated the transport of electrons and reactants/products, which synergistically boosted the electrocatalytic activity of 2D mesoporous metals. Meanwhile, anisotropic 2D mesoporous metals obeyed well the physical Ostwald ripening process and thus enhanced their electrocatalytic stability. Up to date, several important electrochemical reactions, including the oxygen evolution reaction (OER), methanol oxidation reaction (MOR), and hydrogen evolution reaction (HER), have been widely explored with 2D mesoporous metals.

**Fig. 6:**
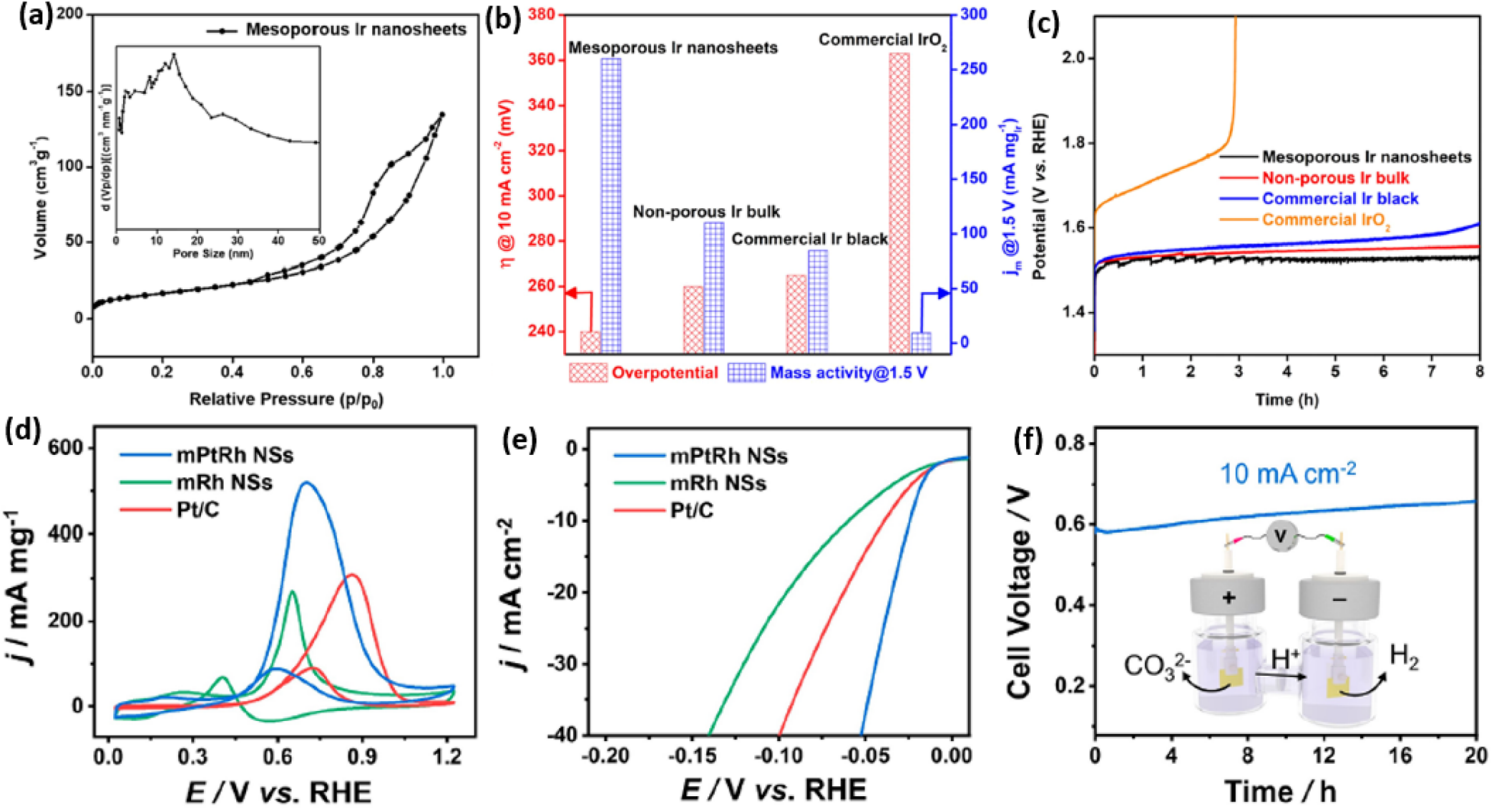
Enhanced catalytic activity and stability. (a) N_2_ adsorption–desorption isotherm plots of mesoporous Ir nanosheets. (b) Summarized overpotentials (at 10 mA cm^−2^) and mass activities (at 1.5 V) and (c) chronopotentiometry stability of mesoporous Ir nanosheets and the counterpart electrocatalysts for OER electrocatalysis. Reproduced from ref. 50 with permission from the American Chemical Society, Copyright 2018. (d) CV curves and (e) LSV curves of mesoporous PtRh nanosheets, mesoporous Rh nanosheets, and commercial Pt/C collected in 1.0 M KOH methanol solution. (f) Long-term chronoamperometry stability of mesoporous PtRh nanosheets collected in 1.0 M KOH methanol solution. Inset in (f) is a schematic illustration of the methanol-assisted H_2_ production process. Reproduced from ref. 64 with permission from the American Chemical Society, Copyright 2022.

We first discuss the electrochemical OER performance, which is the anode reaction of water splitting electrocatalysis, of 2D mesoporous Ir nanosheets as a proof-of-concept reaction in detail.^50^ In comparison to commercial Ir black (20 m^2^ g^−1^), the Brunauer–Emmett–Teller (BET) surface area of mesoporous Ir nanosheets was as high as 42 m^2^ g^−1^, indicating a higher number of metal sites (Fig. 6a). Similarly, a higher electrocatalytic surface area (ECSA) of 88 m^2^ g^−1^ was achieved by mesoporous Ir nanosheets collected in the electrochemical condition. When being performed for EOR electrocatalysis, mesoporous Ir nanosheets disclosed the highest activity with a lowest overpotential (at 10 mA cm^−2^) of 240 mV and a highest mass activity (at 1.5 V) of 260 mA mg^−1^. Remarkably, the OER activity was better than those of its counterparts, non-porous Ir bulk and commercial Ir black, highlighting structural functions in promoting the OER electrocatalysis of mesoporous Ir nanosheets (Fig. 6b). Meanwhile, mesoporous Ir nanosheets were also highly stable in OER electrocatalysis, retaining a constant current density of 10 mA cm^−2^ over 8 h (Fig. 6c).

2D mesoporous metals have also exhibited a higher activity and stability in MOR and HER electrocatalysis. A recent work reported by Wang *et al.* found that mesoporous PtRh nanosheets showed the highest ECSA of 71.4 m^2^ g^−1^, which was remarkably larger than that of the commercial Pt/C catalyst.^64^ Typically, mesoporous PtRh nanosheets disclosed the highest peak current density of 73.6 m^2^ g^−1^ in MOR electrocatalysis and the lowest onset potential of 24 mV (at 10 mA cm^−2^) in HER electrocatalysis (Fig. 6d and e). In sharp contrast, mesoporous Rh nanosheets and Pt/C showed a much lower electrocatalytic activity in the same test conditions, indicating the synergies of a bimetallic PtRh composition and 2D mesoporous nanostructure in promoting electrocatalysis. The authors also demonstrated the high efficiency of mesoporous PtRh nanosheets in the bifunctional electrodes, which performed well in both MOR and HER electrocatalysis at the same time. Meanwhile, chronoamperometry tests showed a slight decrease of current density for 20 h in the bifunctional electrodes, confirming the great potential of mesoporous PtRh nanosheets for practical applications (Fig. 6f). Considering the compositional diversity, 2D mesoporous metals can be further expected to be highly active and stable catalysts in other electrocatalytic reactions and organic catalysis.

### 3.2 Enhanced electrocatalytic selectivity of 2D mesoporous metals

Not only the high activity and stability but also an enhanced selectivity represent the important parameters for evaluating the electrocatalytic performance of electrocatalysts. 2D mesoporous metals have also exhibited a higher selectivity in several electrochemical reactions. First, 2D mesoporous metals ensured more electron-rich active sites along the mesopores, which potentially changed the reaction barriers of the intermediates in electrocatalytic reactions. Second, concave/convex mesochannels of the 2D mesoporous metals provided nanoconfinement microenvironment for intermediates and thereby changed the reaction pathways for some electrocatalytic reactions.

**Fig. 7:**
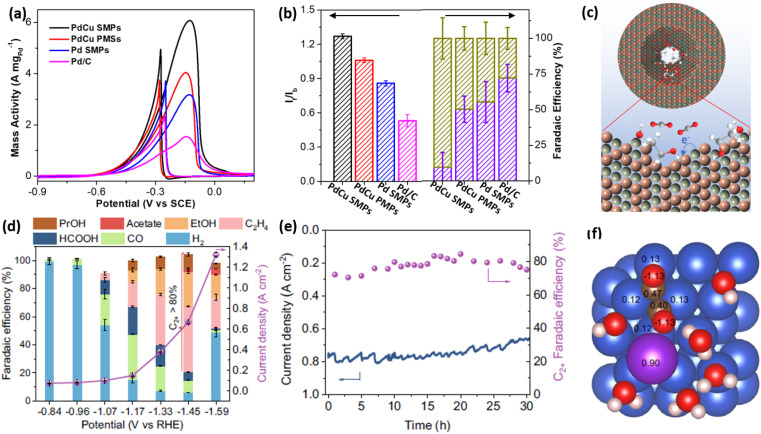
Enhanced catalytic selectivity. (a) EOR activities and (b) summarized *I*_f_/*I*_b_ and FE_C1_ (%) of PdCu single-crystalline mesoporous nanoplates (SMPs) and the counterpart electrocatalysts in an alkaline ethanol solution. (c) Schematic illustration for the EOR mechanism in mesopores. Reproduced from ref. 51 with permission from Wiley, Copyright 2022. (d) Summarized FE_C2+_ and total current densities and (e) long-term stability of mesoporous Cu nanosheets at different potentials during CO_2_RR electrocatalysis. (f) Charge density of *OCCO on a Cu–H_2_O–K^+^ slab based on the Bader charge calculation. Reproduced from ref. 52 with permission from Springer Nature, Copyright 2021.

Ethanol oxidation reaction (EOR) electrocatalysis is a typical multi-step reaction, including the complete electrooxidation to CO_2_ by a 12 electron (12e^−^) reaction pathway and incomplete electrooxidation to acetic acid (CH_3_COOH) by a 4e^−^ reaction pathway.^67–69^ Comparatively, a complete EOR electrocatalysis is more favorable in a direct ethanol fuel cell, since it can produce more electrical energy. Despite great potential, the complete EOR required selective cleavage of a high-energy-barrier C–C bond and thus resulted in a low electrocatalytic selectivity of often less than 20%. Very recently, our group found that 2D single-crystalline mesoporous PdCu nanoplates remarkably promoted the complete EOR electrocatalysis to CO_2_ by the confinement effect of the intermediates.^51^ In an alkaline solution, single-crystalline mesoporous PdCu nanoplates hold the best electrocatalytic mass activity of 6.09 A mg^−1^ for EOR electrocatalysis (Fig. 7a). The mass activity was remarkably higher than those of its counterpart electrocatalysts, including single-crystalline mesoporous Pd nanoplates, polycrystalline mesoporous PdCu nanoplates, and commercial Pd/C, indicating the importance of single-crystallinity and a bimetallic alloy in enhancing EOR activity. Meanwhile, single-crystalline mesoporous PdCu nanoplates disclosed a higher ratio of forward current to backward current (*I*_f_/*I*_b_) of 1.25 and a higher faradaic efficiency of C_1_ products (CO_2_ and CO_3_^2−^) (FE_C1_) of 72.1%, indicating a higher selectivity of complete EOR electrocatalysis (Fig. 7b). Electrochemical EOR kinetic studies further indicated the lower activation energy and higher diffusion rate of single-crystalline mesoporous PdCu nanoplates. Considering the structural functions and alloyed compositions, the higher FE_C1_ of 2D mesoporous PdCu was ascribed to the confinement of an adsorbed OH (Cu–OH*) intermediate within the mesopores that effectively attacked the C–C bond on an adjacent Pd site (Pd(C–C)) and thus promoted the complete electrooxidation of ethanol into CO_2_ (CO_3_^2−^) (Fig. 7c).

The carbon dioxide reduction reaction (CO_2_RR) represented another selective electrocatalytic reaction that can produce high-value chemicals.^4,16,70–73^ By confining the key intermediates within mesopores, 2D mesoporous metal nanosheets not only inhibited its competitive reaction to produce H_2_ but also remarkably increased the FE of C_2+_ (FE_C2+_) products. In the optimized conditions, mesoporous Cu nanosheets hold the highest selectivity for C_2+_ products (ethylene, ethanol, acetate acid, and n-propanol) with a superior FE_C2+_ of 83.7% and a larger partial current density of 0.56 A cm^−2^ (Fig. 7d).^52^ Meanwhile, mesoporous Cu nanosheets were electrocatalytically stable in acidic CO_2_RR, retaining a high current density and FE_C_2+__ after operation for 30 h (Fig. 7e). By contrast, flat Cu nanosheets without mesopores exhibited a much lower FE_C_2+__ of 35.1%. More impressively, mesoporous Cu nanosheets disclosed a higher intrinsic activity of 2.31 mA cm^−2^, which was 22 times higher than that of flat Cu nanosheets (0.11 mA cm^−2^), indicating the importance of penetrated mesopores in promoting CO_2_RR into C_2+_ products. The charge density analysis by calculated Bader charge data demonstrated that, in the presence of K^+^, the key intermediate (*OCCO) was stabilized and further confined in the mesoporous channels of 2D mesoporous Cu nanosheets, which decreased the energy barriers of C–C bond coupling and thus promoted a deeper CO_2_RR electrocatalysis to give C_2+_ products (Fig. 7f). A similar confinement effect of intermediates in Cu hollow cavities had been proposed to enhance FE_C_2+__ in CORR electrocatalysis. Besides, Du *et al.* also found that chemisorption behaviors of CO_2_ in 2D mesoporous Zn nanosheets were different to that on a flat surface, which provided a “nanopincer” effect in concave mesoporous channels.^74^ The “nanopincer” behavior of mesoporous Zn nanosheets lowered the energy barriers of electrocatalysis and thus promoted CO_2_RR to CO. These results clearly highlight that 2D mesoporous metals have high potential for electrocatalysis in not only enhancing activity and stability but also increasing selectivity.

## 4. Conclusion and perspective

Since the first report in 2018, 2D mesoporous metals with well-defined nanostructures have been rapidly advanced. 2D mesoporous metals with different pore structures, elemental compositions, and crystallinity have been achieved by various synthetic strategies and further demonstrated as high-performance electrocatalysts. Compared to other nanostructures, 2D mesoporous metals have featured add-in structural functions, which not only exposed more undercoordinated active sites and accelerated electron/mass transport but also ensured a nanoconfinement microenvironment for reaction intermediates, and thus enhanced activity, stability, and selectivity in electrocatalytic reactions. In this Perspective, we summarized four synthetic strategies of 2D mesoporous metals, including the COI route, HIO route, IG route, and MOAR route, and presented their formation procedures and mechanisms in detail. Meanwhile, we discussed the add-in structural functions of 2D mesoporous metals and further explored their wide utilization as high-performance electrocatalysts, including the enhanced activity and stability as well as increased selectivity. This Perspective is expected to provide the readers a broad guidance for the rational design and synthesis of novel 2D mesoporous metal nanostructures for their utilization in catalysis and electrocatalysis.

Despite much progress, the synthesis and application of 2D mesoporous metals is still in its infancy. There is no doubt that high-performance and functional 2D mesoporous metals that subtly combine structural synergies of asymmetric 2D nanostructure and abundant mesoporosity will continue to flourish. In light of the increasing attention and corresponding challenges in this research topic, we offer some opinions and outlook for future directions in the synthesis and applications of 2D mesoporous metals. On the one hand, 2D mesoporous metals prepared previously were mostly limited to several noble metals (Pd, Pt, and Ir) and their solid alloys. Other compositions (for example, Ru, Rh, their solid alloys, core–shell, and intermetallics) and crystalline phases (for example, hexagonal close packed (hcp), body centered cubic (bcc), and amorphous) have never been reported in 2D mesoporous metals. Meanwhile, the mesostructures of 2D mesoporous metals are mainly disordered and uncontrolled. Therefore, it is highly desirable to develop new synthetic strategies and theories of 2D mesoporous metals with precisely controlled compositions and crystalline phases. On the other hand, the application of 2D mesoporous metals was only reported in several electrocatalytic reactions. Considering the add-in structural functions of 2D mesoporous metals, including desirable optical, electronic, and magnetic properties, it is urgently required to explore their new utilization in other electrocatalytic and photocatalytic reactions and bio-related applications. Furthermore, by means of important characterization techniques and theory calculations, the exploration of structure–property–performance relationships in (electro)catalysis would help the design and synthesis of novel high-performance 2D mesoporous metals. With the efforts of scientists from different directions, we believe that 2D mesoporous metals could open a blue ocean to design a new generation of high-performance functional electrocatalysts in the near future.

## Data availability

All data in the perspective were cited from other references.

## Author contributions

H. L. conceptualized and wrote the perspective. B. L. reviewed and edited the final version of the perspective.

## Conflicts of interest

The authors declare no conflicts of interest.

## Supplementary Material
